# Cost-utility of as-needed ICS-formoterol versus to maintenance ICS in mild to moderate persistent asthma

**DOI:** 10.1186/s12890-021-01775-1

**Published:** 2021-12-05

**Authors:** Jefferson Antonio Buendía, Diana Guerrero Patiño

**Affiliations:** 1 Research Group in Pharmacology and Toxicology ”INFARTO”. Department of Pharmacology and Toxicology, Facultad de Medicina, University of Antioquia, Carrera 51D #62-29, Medellin, Colombia; 2 Hospital Infantil Concejo de Medellin, Medellin, Colombia

**Keywords:** Budesonide-formoterol, Uncontrolled asthma, Cost-effectiveness analysis, Decision analysis, Markov model

## Abstract

**Background:**

Recent asthma guidelines, such as the Global Initiative for Asthma (GINA), recommend in adult patients as-needed inhaled corticosteroids (ICS)-formoterol as an alternative to maintenance ICS in mild to moderate persistent asthma. The introduction of these recommendations concerns whether using as-needed budesonide-formoterol would be more cost-effective than to maintenance ICS. This study aimed to evaluate the cost-effectiveness of as-needed combination low-dose budesonide-formoterol compared to short-acting β2-agonist (SABA) reliever therapy in patients with mild asthma.

**Methods:**

A probabilistic Markov model was created to estimate the cost and quality-adjusted life-years (QALYs) of patients with mild asthma in Colombia. Total costs and QALYs of low-dose budesonide-formoterol compared to short-acting β2-agonist (SABA) were calculated over a lifetime horizon. Multiple sensitivity analyses were conducted. Cost-effectiveness was evaluated at a willingness-to-pay value of $19,000.

**Results:**

The model suggests a potential gain of 0.37 QALYs and per patient per year on as-needed ICS-formoterol and a reduction in the discounted cost per person-year, of as-needed ICS-formoterol to maintenance ICS, of US$40. This position of dominance of as-needed ICS-formoterol negates the need to calculate an incremental cost-effectiveness ratio. In the deterministic and probabilistic sensitivity analysis, our base‐case results were robust to variations in all assumptions and parameters.

**Conclusion:**

Low-dose budesonide-formoterol as a reliever was cost-effective when added to usual care in patients with mild asthma. Our study provides evidence that should be used by decision-makers to improve clinical practice guidelines and should be replicated to validate their results in other middle-income countries.

## Introduction

Asthma is an obstructive respiratory disease more prevalent around the world [1]. Their incidence is growing, especially in developing countries due to, an increase in, among others, causes of, prompt diagnosis, and improved health services access. However, still, not all patients are classified y correctly treated according to their asthma severity [2]. This generates a high burden of diseases and costs [3]. For example, the median cost of uncontrolled asthma per patient is three times higher than the cost of mild asthma [4]. Additionally, this cost would be higher if we added the cost due to the reduced productivity of patients with uncontrolled asthma [5]. Severe asthma is a serious problem for health systems [6]. In this sense, having drugs that achieve the most effectiveness at the lowest cost possibly becomes a priority for the health system worldwide.

Recent asthma guidelines, such as the Global Initiative for Asthma (GINA), recommend in adult patients as-needed ICS-formoterol as an alternative to maintenance ICS in mild to moderate persistent asthma [7]. A recent meta-analysis, of four randomized controlled trials with 8065 participants, reveals as-needed, ICS-formoterol was associated with fewer ED visits in the as-needed budesonide-formoterol group (POR 0.65; 95% CI 0.43–0.98) without difference in serious adverse events (OR 1.07; 95% CI 0.84–1.36) [8–10]. Also, real world studies have reported that, as-needed, budesonide-formoterol reduced the risk of severe exacerbation compared with maintenance budesonide plus SABA reliever in patients with mild to moderate asthma [11].

The introduction of these recommendations concerns whether using as-needed budesonide-formoterol would be more cost-effective than to maintenance ICS. This question is even more relevant in developing countries due to the increasing prevalence of asthma and constrained healthcare costs in most countries. An economic evaluation of these new drugs could provide evidence to optimize the efficiency of the use of economic resources in these countries. This study aimed to assess the health and economic consequences of as-needed ICS-formoterol in mild to moderate persistent asthma.

## Materials and methods

### Model structure

**Fig. 1:**
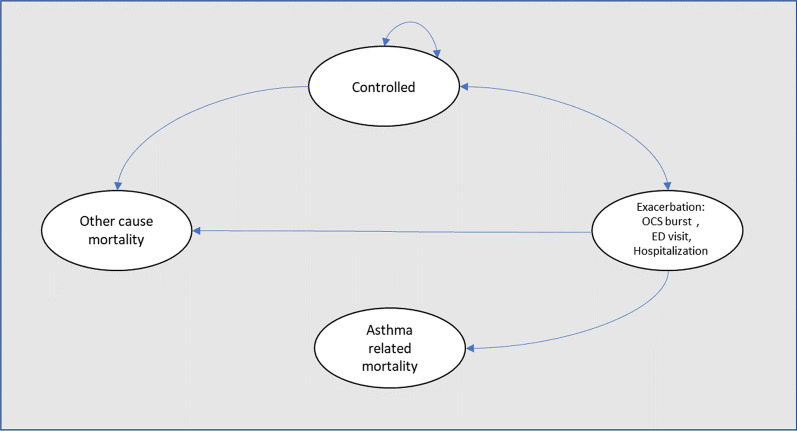
Markov model

We conducted a probabilistic Markov model to estimate the cost and quality-adjusted life-years (QALYs) associated with “as-needed” ICS-formoterol and maintenance ICS in mild to moderate persistent asthma. In this probabilistic model, a cohort of patients could transition between four mutually exclusive health states (symptom-free state or asthma-controlled, asthma exacerbation, asthma-related mortality, and all-cause mortality). In asthma exacerbation, there are three levels of exacerbations: OCS burst (which was defined as relatively major symptoms during the week and need of use of oral corticosteroids to achieve the control of symptoms), emergency department or ED (patient that request treatment with systemic corticosteroids), and hospitalization. Asthma-related mortality may occur only after exacerbation with hospitalization, while all-cause mortality may occur from any health state (Fig. 1). We made this analysis from a societal perspective (including direct and indirect costs), using a lifetime horizon and a cycle length of 4 weeks. Half-cycle correction and an annual discounting rate of 5% were applied to both costs and benefits in the base case. Treatment was considered cost-effective if the incremental cost-utility ratio was below $19.000 per QALY gained using the World Health Organization (WHO) recommendation of three times the GDP per capita to define the willingness to pay (WTP) in Colombia.

### Parameters of the Markov model

Multiple parameters were derived from published research and local data, which are presented in Table 1. Data of relative risk (RR) on exacerbation rates were extracted from a recent meta-analysis of four RCTs (n = 8065 participants). In this study, this combination reduced the rate ratio of severe exacerbations (RR 0.85; 95% CI 0.72–1.00) and ED visits (OR 0.65; 95% CI 0.43–0.98) [9]. The transition probabilities for moving between different health states of the standard therapy and add-on therapy were derived from previous clinical trials of as-needed budesonide–formoterol in mild asthma [12]. Data of utilities of each Markov state were extracted from a systematic review of utilities in asthma [13, 14]. Since all these data (RR, transition probabilities, and utilities) do not come from the Colombian population, they were subjected to probabilistic sensitivity analysis as detailed below, and as recommended by Consolidated Health Economic Evaluation Reporting Standards (CHEERS) Statement [15]. In this sensitivity analysis, to build the range of RR to be used in this analysis, we use the CI 95% of RR published by clinical trials [8–10]. In the case of utilities and transition probabilities, the upper and lower ranges were estimated by adding or subtracting 25% of the value from the central value defined for the base case. The risk of asthma mortality and mortality from other causes was estimated using age- and gender-specific Colombian life tables mortality (2016 to 2020) [16].

**Table 1:** Base case

Variable	Base case	Valor high	Valor low	References
Cost US$				
Cost Budesonide-Formoterol (per 120 doses)	$53	$66	$40	
Cost controlled (anual)	$416	$520	$312	[17]
Cost mild exacerbation (per episode)	$94	$118	$71	
Cost moderate exacerbation (per episode)	$191	$239	$143	
Cost severe exacerbation (per episode)	$386	$483	$290	
Utilities (anual)				
Controlled asthma	0.92	1	0.69	
Exacerbation with OCS Burst	0.86	1	0,65	[13]
Exacerbation with ED visit	0,83	1	0,62	
Exacerbation with Hospitalization	0.74	0.93	0.56	
Budesonide + Formoterol efect				
Relative risk on exacerbation rate	0.85	0.72	1	
Relative risk on ED visit	0.65	0.43	0.98	[9]
Adherence to as need—budesonide-formoterol	68%	85%	51%	[10]
Adherence to IC daily	62%	78%	47%	[10]
As-Needed budesonide-formoterol by Time of Day	0.52	0.65	0.39	
Transition probabilities				
Probability controlled to OC Burst	0.11	0.14	0.10	
Probability OCS Burst to ED visit	0.02	0.03	0.02	[12]
Probability of ED visti to hospitalization	0.0117	0.01	0.01	
Asthma mortality	0.0000020	0.00	0.00	
Annual dicount rate	5%	6%	0%	

All costs of each health state defined in the Markov model were extracted from a previously published Colombian-based study [17]. Briefly, this study identified the asthma-related direct and indirect costs of 1131 patients with severe asthma from January 1, 2004, through December 31, 2014, in Colombia. Asthma severity classification was mainly based on the paper of Jacob et al. [18]. Mild persistent asthma in this cost study was defined using according to the definition of mild asthma of GINA and according to SABA consumption (six SABA fills and zero oral OCS fills per year, or two to three SABA fills and less than two OCS fills per year, or one exacerbation). This criterion related to using rescue medication per year may be more accurate than using LABA and ICS given the high frequency of underuse and prescription of controller medications in Latin American countries [19]. Unit drug costs were taken from the National Drug Price Information System (SISMED, 2020). All cost costs were transformed to 2020 costs using official inflation data in Colombia. We use US dollars (Currency rate: US$1.00 = COP$ 3,000) to express all costs in the study [16].

### Sensitivity analysis

To explore parameter uncertainty of the model inputs, first, we conducted a one-way sensitivity analysis represented in a tornado diagram. Also, we performed probabilistic sensitivity analysis by randomly sampling from each of the parameter distributions (beta-distribution in the case of relative risk and utilities; Dirichlet distribution for multinomial data in the case of transition probabilities, and gamma distribution in the case of costs). The expected costs and expected QALYs for each treatment strategy were calculated using that combination of parameter values in the model. This process was replicated one thousand times (i.e., second-order Monte Carlo simulation) for each treatment option resulting in the expected cost-utility. All analyses were done in Microsoft Excel®.

## Results

### Case based analysis

The base-case analysis showed that as-needed ICS-formoterol compared to maintenance ICS in mild to moderate persistent asthma SABA. This combination was associated with lower cost higher QALYs. The main results are presented in Table 2. In the analysis of the Markov cohort model, we estimated a median probability of survival free of exacerbation of 0.63 in as-needed ICS-formoterol 0.62 in maintenance ICS.

**Table 2:** Cost-effectiveness of as-needed Budesonide-formoterol

Strategy	Cost	Marginal difference	QUALYs	Marginal difference	C/E	ICER
As-needed Budesonide-formoterol	$71		4.2		$17	
Maintenance ICS	$106	-$36	3.8	0.368	$28	Dominated

The model suggests a potential gain of 0.37 QALYs and per patient per year on as-needed ICS-formoterol in the deterministic model and 0.21 QALYs in the probabilistic model. For as-needed ICS-formoterol, the total discounted cost per person-year was US$35 in the deterministic model and US$ 29 in the probabilistic model. The model suggests a potential reduction in the discounted cost per person-year, of as-needed ICS-formoterol concerning maintenance ICS, of US$40 in the deterministic model and US$30 in the probabilistic model. This position of dominance of as-needed ICS-formoterol negates the need to calculate an incremental cost-effectiveness ratio.

### Sensitivity analyses

**Fig. 2:**
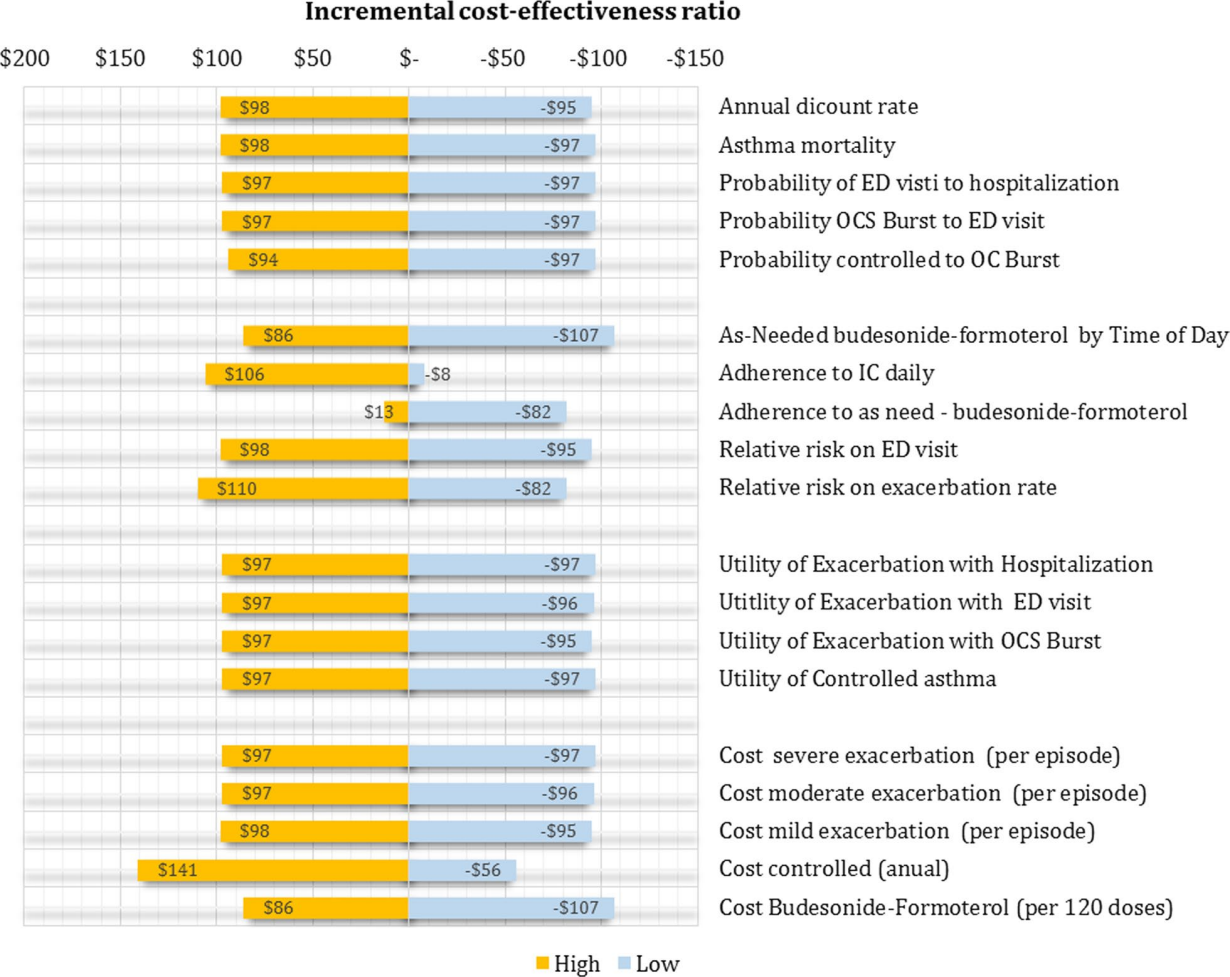
Tornado diagram

**Fig. 3:**
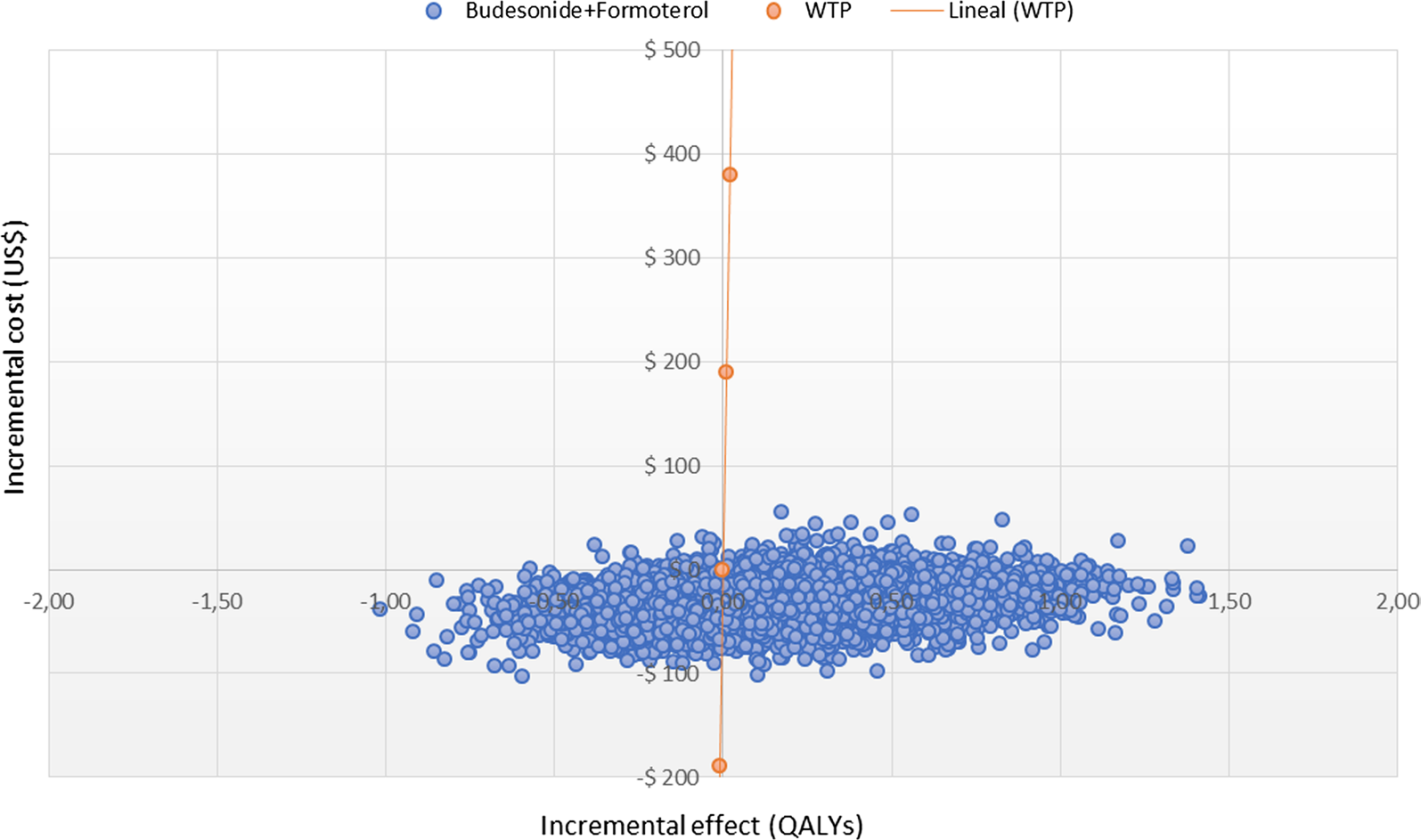
Cost effectiveness plane

**Fig. 4:**
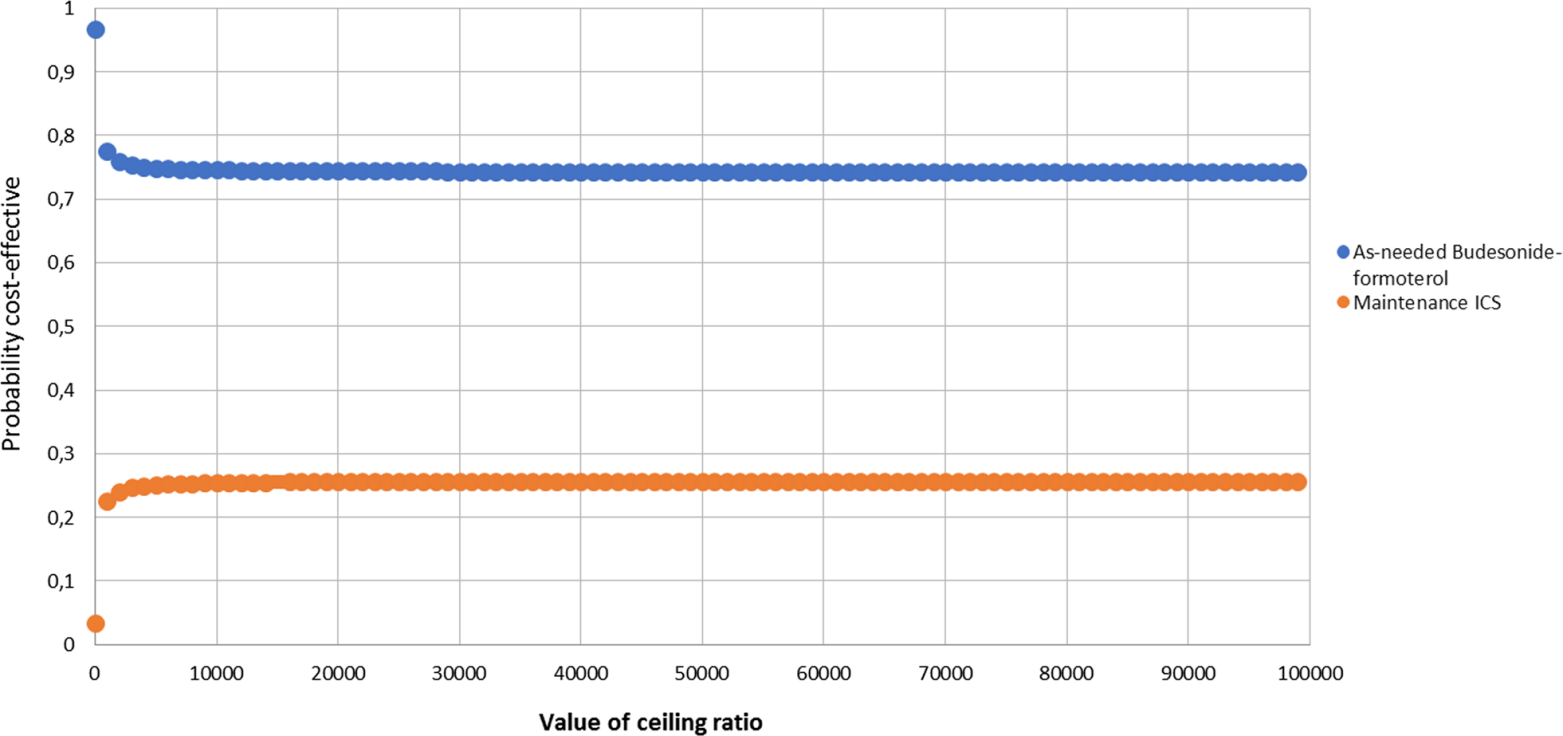
Cost-effectiveness acceptability curve

In the deterministic sensitivity analysis, our base‐case results were robust to variations of all assumptions and parameters, including pharmacological adherence to the two evaluated strategies. For none of the variables evaluated, variations within the established ranges led to the incremental cost-effectiveness ratio being higher than the WTP, Fig. 2. The results of probabilistic sensitivity analysis are graphically represented in the cost-effectiveness plane, Fig. 3. This scatter plot shows that compared with SOC, treatment with azithromycin tends to be associated with lower costs and higher QALY. Indeed, 86% of simulations were graphed in quadrant 2 (lower cost, high QALYs), 1.5% in quadrant 1 (high cost, high QALYs), 12% in quadrant 3 (lower cost, lower QALYs), and 0.5% in quadrant 4 (high cost, lower QALYs). The cost-effectiveness acceptability curve shows that low-dose budesonide-formoterol becomes cost-effective in 100% for all willingness-to-pay thresholds, Fig. 4.

## Discussion

Our study showed that as-needed ICS-formoterol compared to maintenance ICS in mild to moderate persistent asthma SABA, this combination was associated with lower cost and higher QALYs. These findings complement and support the GINA 2021 recommendation that as-needed ICS-formoterol is an alternative to maintenance ICS at step 2, being this combination is not only effective but also therapeutic efficient.

To our knowledge, this is the first economic evaluation of this combination in mild asthma. Our results are in line with the results of effectiveness estimates in previously meta-analysis. Hatter et al., show the result of a pooled analysis of four RTC with 8065 participants. In this study, as-needed, ICS-formoterol was associated with a prolonged time-to-first severe exacerbation (hazard ratio 0.85; 95% CI 0.73–1.00; p = 0.048) and reduced daily ICS dose (mean difference − 177.3 μg, 95% CI − 182.2– − 172.4 μg)[9]. This can be explained by the ability of patients to take as-needed budesonide-formoterol before it becomes severe. Patients on maintenance ICS are restricted to fixed twice-daily dosing and this can delay the prompt use of greater doses of ICS and reliever to abort the exacerbation. Our study finds differences not only in event-free survival probabilities during mathematical modeling but also in QALYs; being concordant with the results of efficacy in clinical trials.

In our results for both the probabilistic and deterministic models, the difference in QALYs was relatively small. The differences were in the deterministic model 0.37 and the probabilistic model 0.21. This is a relevant finding that only economic evaluations can reveal. Although in clinical trials the reductions in risk of exacerbations of a relevant magnitude (greater than 35%), this does not necessarily translate into a bigger increase in life-years gained or quality-adjusted life-years gained. Aspects that for the moment can only be estimated by mathematical simulations, since long follow-up periods and large sample sizes would be needed in clinical trials to demonstrate differences in survival or not.

These estimates are reliable, given that our model was robust to variations in earnings, transition probabilities, and costs. Indeed, we decided to use utilities reported in a systematic review to have broader values and in more diverse populations. Variations in the values of these utilities in the probabilistic sensitivity analysis did not significantly change the calculated ICER. Indeed, after 10 000 simulations in our probabilistic sensitivity analysis as-needed, combination low-dose budesonide as a reliever tends to be associated with an ICER below of WTP.

A crucial methodological aspect is discussing willingness to pay (WTP) to declare Colombia a cost-effective technology or not. Since Colombia does not have a threshold that represents the WTP per unit of effectiveness (QALY), the ICER results per QALY were evaluated by using the reference corresponding to the World Health Organization (WHO) recommendation (three times the GDP per capita). Not having an own estimate of the WTP may be debatable; however,, up to now, all the economic evaluations in health carried out in the country follow the threshold suggested by the WHO, which has also been endorsed by the national technology evaluation agency [20–24]. The results of the probabilistic sensitivity analysis confirm the robustness of the model results. Since transition probabilities and utilities do not come from the Colombian population, they were subjected to probabilistic sensitivity analysis as detailed below as recommended by Consolidated Health Economic Evaluation Reporting Standards (CHEERS) Statement [15].

Our study has some limitations. We used utilities extracted from the literature and not estimated directly from our population. As was mentioned previously, the reliability and robustness of the results were evaluated by sensitivity analysis. Our result only refer to patient with mild asthma and cannot be extrapolated to patients with the use of daily corticosteroids.

## Conclusion

In conclusion, low-dose budesonide-formoterol as a reliever was cost-effective when added to usual care in patients with mild asthma. This evidence should be used by decision-makers to improve clinical practice guidelines and should be replicated to validate their results in other middle-income countries.

## Data Availability

DB SMART Step 2 [Data set]. Zenodo. http://doi.org/10.5281/zenodo.4990162.

## Abbreviations

ICS: Inhaled corticosteroids
LABA: Long-acting beta2-agonist
OCS: Oral corticosteroids
LAMA: Long-acting muscarinic antagonists
QALYs: Quality-adjusted life-years
SOC: Standard therapy
WTP: Willingness to pay
RR: Relative risk
CHEERS: Consolidated health economic evaluation reporting standards
SABA: Short-acting beta-agonists
ICER: Incremental cost-effectiveness rate
AMR: Antimicrobial resistance
